# Chronic Hypoxia-Induced Reprogramming of Smooth Muscle Cells: Insights into Transcription Factors, Cellular Stresses, and Therapeutic Implications

**DOI:** 10.3390/biomedicines14081843

**Published:** 2026-08-16

**Authors:** Sheng-Huei Wang, Yuan-Ming Tsai, Shu-Ting Liu, Shih-Ming Huang

**Affiliations:** 1 Institute of Medical Sciences, College of Medicine, National Defense Medical University, Taipei 11490, Taiwan; slidersinker@gmail.com; 2 Division of Pulmonary and Critical Care Medicine, Department of Internal Medicine, Tri-Service General Hospital, National Defense Medical University, Taipei 11490, Taiwan; 3 Division of Thoracic Surgery, Department of Surgery, Tri-Service General Hospital, National Defense Medical University, Taipei 11490, Taiwan; 4 Institute of Biochemistry, College of Biomedical Sciences, National Defense Medical University, Taipei 11490, Taiwan; shuting0719@gmail.com

**Keywords:** hypoxia, hypoxia-inducible factor-1 alpha, reactive oxygen species, angiotensin II, quiescence

## Abstract

**Background/Objectives:** Chronic hypoxia promotes a shift in smooth muscle cells from a quiescent, contractile state to a highly proliferative phenotype, contributing to persistent vascular remodeling. This study examines primary human esophageal smooth muscle cells (HEsSMCs) exposed to hypoxia mimics, desferoxamine (DFO) and dimethyloxallyl glycine (DMOG), and their interaction with angiotensin II (AngII). **Methods:** We utilized HEsSMCs to assess the effects of DFO, DMOG, and AngII through MTT metabolic activity assays, Western blotting analyses, and flow cytometry to evaluate various cellular functions. Additionally, we reanalyzed the public RNA sequencing dataset GSE193817 to explore the contextual influences of DFO-induced responses in HEsSMCs. **Results:** Our findings indicate that both DFO and DMOG stabilize hypoxia-inducible factor-1 alpha proteins at different time intervals, revealing distinct cell cycle profiles and varied protein responses. DFO was shown to increase mitochondrial reactive oxygen species (ROS) while simultaneously suppressing cytosolic ROS, suggesting the involvement of multiple pathways beyond those activated by hypoxic stress. Flow cytometry analysis revealed that DFO triggers late-stage apoptosis, along with increases in overall hypoxia, ROS, autophagy, lipid peroxidation, and mitochondrial membrane depolarization in HEsSMCs. Furthermore, AngII was observed to inhibit the activation of nuclear factor-κB induced by DFO. The analysis of the GSE193817 dataset illuminated the transitional dynamics between quiescent and proliferative states in smooth muscle cells, indicating that AngII signaling is modular and does not simply counteract DFO-induced effects. **Conclusions:** This research enhances our understanding of hypoxia and ROS responses in HEsSMCs and underscores the potential for personalized medicine informed by sequencing data.

## 1. Introduction

Smooth muscle contraction plays a crucial role in various homeostatic functions, including the regulation of blood pressure, gastrointestinal motility, and the behavior of the urogenital tract [1]. Recent studies have shown that many smooth muscle types are significantly influenced by hypoxia on a rapid time scale. The impact of hypoxia on contraction can have beneficial effects, such as the dilatation of blood vessels, which increases blood flow to hypoxic regions, or the preservation of ATP during metabolic stress. Hypoxia can be categorized into three types: acute hypoxia (lasting from minutes to hours), chronic hypoxia (extending from days to years), and intermittent hypoxia (characterized by alternating periods of hypoxia and normoxia) [2]. In cases of chronic hypoxia, the master transcription factor hypoxia-inducible factor-1 alpha (HIF-1α) becomes stabilized [3]. This stabilization triggers a phenotypic switch in smooth muscle cells from a contractile state to a synthetic/proliferative state [4]. As chronic hypoxia forces smooth muscle cells out of their quiescent, contractile state, it transforms them into highly proliferative and secretory cells, serving as a fundamental driver for chronic vascular remodeling [4]. Thus, while hypoxia can initially promote beneficial adaptations, it can also lead to dysfunction, which has been linked to various disease states, including pulmonary arterial hypertension, atherosclerosis, vascular restenosis, asthma, airway remodeling, and uterine dystocia [5]. In essence, hypoxia not only injures smooth muscle cells but also reprograms them toward a pathogenic phenotype, thereby contributing to tissue remodeling and creating a microenvironment that supports disease and cancer progression.

Cellular hypoxia, characterized by oxygen levels between 0.5% and 2%, can be either transient, resulting from temporary mismatches between oxygen supply and metabolic demands, or chronic, stemming from permanent vascular inadequacies, unresolved tissue edema, and inflammation [3,6]. All these adaptations must work in concert to support critical cellular functions until both tissue and organismal responses restore oxygen levels to normal. Hypoxia exerts multiple effects on the vascular system, with the primary molecular sensors being HIFs and heme oxygenase. A pivotal advancement in our understanding of cellular responses to fluctuations in oxygen levels was the discovery of HIFs and their regulatory mechanisms involving the von Hippel–Lindau (VHL) tumor suppressor protein (pVHL) and prolyl hydroxylases (PHDs), which are members of the α-ketoglutarate (α-KG) dioxygenase superfamily [3]. Hypoxia triggers a substantial transcriptional induction of genes governed by HIFs, along with a variety of other transcription factors, including nuclear factor-κB (NF-κB) [7]. Despite this diversity, the majority of oxygen-sensitive genes are direct targets of HIFs, playing crucial roles at both cellular and organismal levels in adapting to declining oxygen availability. However, sustained activation of hypoxia-induced genes can lead to pathological conditions, such as pulmonary hypertension.

The mitochondrial electron transport chain (ETC) is the primary consumer of intracellular oxygen for ATP generation through oxidative phosphorylation in most cell types [8]. Consequently, fluctuations in oxygen levels are likely to impact the activity of the mitochondrial ETC. Hypoxia influences ETC function at various levels, including the regulation of different mitochondrial ETC complexes and the availability of reducing equivalents from the TCA cycle, specifically NADH and FADH_2_ [3,8]. One significant advantage of hypoxia-induced reductions in respiratory rates is the corresponding decrease in the production of mitochondrial reactive oxygen species (ROS) [9]. While low levels of ROS can initiate essential cellular signaling events, elevated ROS levels can lead to cellular injury [10]. Importantly, despite a global shutdown of translation during hypoxia, not all mRNA translation is completely halted, which is vital for cell survival under low oxygen conditions [11].

Reprogramming of intracellular metabolism is a well-documented response to oxygen deprivation [12]. One of the most notable adaptations observed in oxygen-starved cells is the increased uptake of glucose and heightened glycolytic flux, driven by various HIF-1 target genes to facilitate glucose catabolism [3,13]. This increase is particularly pronounced under severe hypoxic conditions (approximately 0.1–1% oxygen) across different cell lines and supports cell survival by promoting hypoxia-induced autophagy [14]. In such severe hypoxia, which often coincides with significant nutrient depletion, autophagy can be triggered through HIF-independent mechanisms. This process enables the removal of damaged organelles and the release of nutrients that are critical for maintaining cell viability, thereby enhancing survival in stressful environments [15]. However, activation of HIF alone is insufficient to reverse disease states; thus, alternative strategies that either reduce oxygen delivery or modulate more specific pathways downstream of hypoxia are essential.

To prevent the reoxygenation of smooth muscle cells, we employed chemical hypoxia mimics such as DFO (Desferoxamine) and DMOG (Dimethyloxallyl Glycine). These compounds target PHD enzymatic activity by chelating iron and mimicking α-ketoglutarate, respectively [3,16]. Hypoxic stress in mitochondria triggers a surge of ROS, particularly through the activation of NADPH oxidase 4 [17]. This oxidative stress subsequently activates the MAPK/ERK and Akt/mTOR signaling pathways, enhancing autocrine and paracrine loops that accelerate tissue remodeling and recruit immune cells. Under hypoxic conditions, stabilized HIF-1α binds directly to the promoter of Angiotensin-Converting Enzyme (ACE), resulting in a marked upregulation of its transcription [18]. Simultaneously, hypoxia downregulates ACE2, the enzyme responsible for degrading angiotensin II (AngII) into the vasodilatory peptide Ang-(1-7), which then binds to the Mas receptor on smooth muscle cells [19]. This interaction provides a direct anti-proliferative, anti-fibrotic, and vasodilatory signal, effectively serving as an endogenous rescue mechanism against hypoxia and AngII-induced damage. In clinical practice, targeting the AngII pathway has emerged as one of the most successful and widely adopted therapeutic strategies in modern medicine [20,21]. AngII exerts its effects primarily through interaction with the AngII receptor (AT1R), which activates downstream signaling cascades, leading to a significant increase in intracellular ROS production [20,22,23]. Since AngII promotes smooth muscle cell proliferation, vasoconstriction, and tissue fibrosis, inhibiting this pathway is crucial for halting the progression of cardiovascular and tissue remodeling diseases.

In this study, we utilized primary human esophageal smooth muscle cells (HEsSMCs) to investigate the effects of chemical hypoxia mimics, specifically DFO and DMOG, and to explore the potential interactions between hypoxia and AngII. Additionally, we reanalyzed the publicly available RNA sequencing dataset GSE193817 to provide an orthogonal transcriptomic context for the DFO-induced phenotypes observed in HEsSMCs. Our findings offer new insights into how cellular hypoxia and ROS induced by various environmental stressors may exhibit different levels of responsiveness in HEsSMCs. In clinical settings, personalized medicine that incorporates detailed sequencing information has the potential to enhance therapeutic efficacy in the future.

## 2. Materials and Methods

### 2.1. Cell Culture and Reagents

Human esophageal smooth muscle cells (HEsSMCs) were obtained from ScienCell Research Laboratories (Carlsbad, CA, USA), catalog number 2710, and cultured in Smooth Muscle Cell Medium (SMCM, Cat. #1101) supplemented with Smooth Muscle Cell Growth Supplement (SMCGS, Cat. #1152), 10% (*v*/*v*) fetal bovine serum (FBS, Cat. #0010), and 1% (*v*/*v*) penicillin/streptomycin solution (P/S, Cat. #0503). Poly-L-lysine-coated culture vessels (2 mg/cm^2^) were prepared one day before subculturing. DFO (Desferoxamine mesylate, D9533), angiotensin II (AngII), thiazolyl blue tetrazolium bromide (MTT), propidium iodide (PI), 2′,7-dichlorofluorescein diacetate (DCFH-DA), and acridine orange (Cat. No. A8097) were purchased from Sigma Aldrich (St. Louis, MO, USA). Dimethyloxallyl glycine (DMOG, Cat. No.: HY-15893) was obtained from MedChemExpress LLC (Monmouth Junction, NJ, USA).

### 2.2. Metabolic Activity Analysis

HEsSMCs were plated in a 96-well culture plate at a density of 2.5 × 10^4^ cells per well and treated with the specified concentrations of DFO and DMOG the following day for 24 h. After treatment, the cells were incubated with MTT solution for 1 h at 37 °C. Subsequently, dimethyl sulfoxide was added, and the absorbance was measured at 570 nm and 650 nm using a multimode microplate reader (Varioskan™ LUX, Thermo Scientific™, Waltham, MA, USA). The metabolic activity of the cells was calculated based on the absorbance ratio between the treatment groups and the control groups, with the control defined as 100% cell metabolic activity.

### 2.3. Flow Cytometry Analysis for Cell Cycle Profile, Cell Proliferation, Apoptosis, ROS, Lipid Peroxidation, Autophagy, and Mitochondrial Membrane Potential (MMP)

Cell cycle profiles were assessed by measuring cellular DNA content using flow cytometry. Briefly, HEsSMCs were seeded in 6-well culture plates and treated with the specified concentrations of DFO and DMOG for 24 h. Subsequently, the cells were fixed in 70% ice-cold ethanol and stored at −30 °C overnight. After fixation, they were washed twice with ice-cold PBS supplemented with 1% FBS and stained with propidium iodide (PI) solution (5 μg/mL PI in PBS, 1% Tween 20, and 0.5 μg/mL RNase A) for 30 min at 37 °C in the dark. Finally, the stained cells were analyzed using a FACSCalibur flow cytometer and CellQuest Pro software (version 5.1, BD Biosciences, Franklin Lakes, NJ, USA).

Cell proliferation was evaluated using immunofluorescent staining with bromodeoxyuridine (BrdU) (BD Pharmingen™ BrdU Flow Kit) and analyzed via flow cytometry, following the manufacturer’s instructions. Briefly, the cells were seeded in 6-well culture plates and treated with the specified concentrations of DFO for 24 h. After incubation, the cells were stained with BrdU, harvested, and washed with PBS. They were then fixed and permeabilized before being stained with fluorescent antibodies specific to BrdU. The cells were resuspended in 7-AAD solution to stain total DNA for cell cycle analysis. Finally, the cells were suspended in a staining buffer, and fluorescence analysis for FITC-BrdU was performed using a FACSCalibur flow cytometer and CellQuest Pro software (BD Biosciences).

Early and late stages of apoptosis in cells were assessed using a fluorescein phycoerythrin (PE)-Annexin V Apoptosis Detection Kit (BD Biosciences). According to the manufacturer’s protocol, the cells were stained with PE-Annexin V and 7-Amino-Actinomycin (7-AAD) to evaluate the effects of the specified concentrations of DFO on early apoptosis (PE-Annexin positive and 7-AAD negative), late apoptosis (PE-Annexin positive and 7-AAD positive), and necrosis (PE-Annexin negative and 7-AAD positive). The stained cells were then analyzed using a FACSCalibur flow cytometer and CellQuest Pro software (BD Biosciences).

Intracellular and mitochondrial ROS levels were quantified using the fluorescent markers DCFH-DA and MitoSOX-Red, respectively. Cells were treated with various concentrations of DFO and DMOG for 24 h and then stained with DCFH-DA (10 μM) or MitoSOX-Red for 40 min at 37 °C. After harvesting, the cells were washed once with PBS, and fluorescence was analyzed on channels FL-1 (for DCFH-DA) and FL-2 (for MitoSOX-Red) using a FACSCalibur flow cytometer and CellQuest Pro software (BD Biosciences). The cell volume gating strategy utilized forward scatter height (FSC-H) and side scatter height (SSC-H), with the median fluorescence intensity of the control (vehicle) serving as the reference point for M2 gating.

The status of cell lipid peroxidation was assessed using the BODIPY™ 581/591 C11 fluorescent dye (D3861, Thermo Scientific™, Waltham, MA, USA). Cells were treated with various concentrations of DFO for 24 h and then harvested. After harvesting, the cells were stained with BODIPY-C11 581/591 (10 μM) for 30 min at 37 °C. Following a wash with PBS, fluorescence intensity was analyzed on channel FL-1 of the FACSCalibur flow cytometer using CellQuest Pro software (BD Biosciences). In its reduced state, BODIPY-C11 exhibits excitation and emission maxima at 581/591 nm; upon oxidation, the fluorescence shifts to excitation and emission maxima of 488/510 nm.

Autophagy was assessed by detecting acidic compartments within the cells using acridine orange (AO) staining, followed by analysis via flow cytometry. The protonated form of acridine orange accumulates in acidic vesicles, serving as a marker for the final stages of the autophagy process. Briefly, cells were treated with the specified doses of DFO for 24 h, stained with acridine orange (1 μg/mL) for 20 min at 37 °C, and then trypsinized for harvesting. Afterward, the cells were washed with PBS, resuspended in 400 μL of PBS, and analyzed using a FACSCalibur flow cytometer (BD Biosciences). The excitation wavelength was set at 488 nm, and fluorescence was detected at 510–530 nm (green fluorescence, FL1) and 650 nm (red fluorescence, FL3). The data were analyzed with CellQuest™ software(version 5.1, BD Biosciences), and the percentage of autophagic cells was calculated based on the cell counts in the upper-left and upper-right quadrants.

Mitochondrial depolarization was assessed by measuring the decrease in the red/green fluorescence intensity ratio. All viable and dead cells were harvested, washed with PBS, and incubated with 1× binding buffer containing the MMP-sensitive fluorescent dye JC-1 (BD™ MitoScreen) for 30 min at 37 °C in the dark. After a single wash with PBS, JC-1 fluorescence was analyzed on channels FL-1 and FL-2 of the FACSCalibur flow cytometer using CellQuest Pro software (BD Biosciences) to detect the monomeric (green fluorescence) and aggregated (red fluorescence) forms of the dye.

### 2.4. Hypoxia/Oxidative Stress Analysis

HEsSMCs were treated with various concentrations of DFO for 24 h and then stained with Hypoxia Red and a ROS detection dye (ENZ-51042, Enzo Life Sciences, Long Island, NY, USA) for 30 min at 37 °C, following the manufacturer’s protocol. After staining, cellular hypoxia levels were quantified on the FL-3 channel, while ROS levels were assessed on the FL-1 channel using a FACSCalibur flow cytometer (BD Biosciences). The data was analyzed using CellQuest Pro software.

### 2.5. Western Blotting Analysis

HEsSMCs were lysed using radioimmunoprecipitation assay buffer consisting of 100 mM Tris-HCl (pH 8.0), 150 mM NaCl, 0.1% SDS, and 1% Triton-X 100 to extract proteins. The protein concentration was quantified using a DC protein assay, and 30 µg of protein was loaded onto an SDS-PAGE gel. After electrophoresis, the proteins were transferred to polyvinylidene fluoride membranes (Immobilon-P; Millipore, Bedford, MA, USA), which were then blocked with 5% (*w*/*v*) nonfat milk in TBST for 1 h. Subsequently, the membranes were incubated with primary antibodies overnight at 4 °C with gentle shaking, followed by a 1-h incubation with secondary antibodies at room temperature. Primary antibodies against NFκB-p65 (catalog no.: sc-372, 1:1000 dilution), p62 (catalog no.: sc-28359, 1:1000 dilution), Cyclin B1 (catalog no.: sc-245, 1:1000 dilution), GAPDH (catalog no.: 47724, 1:2000 dilution), and α-actinin (ACTN, catalog no.: sc-17829, 1:5000 dilution) were obtained from Santa Cruz Biotechnology (Santa Cruz, CA, USA), and primary antibodies against HIF-1α (catalog no.: #14179, 1:1000 dilution), p-mTOR (catalog no.: 2971, 1:1000 dilution), mTOR (catalog no.: 2972, 1:1000 dilution), p-Akt (catalog no.: #9271, 1:1000 dilution), Akt (catalog no.: #4691, 1:1000 dilution), p-ERK1/2 (catalog no.: 4370, 1:1000 dilution), ERK1/2 (catalog no.: 4695, 1:2000 dilution), H3 (catalog no.: #9715, 1:3000 dilution), LC3B (catalog no.: #2775, 1:1000 dilution), and p-NFκB-p65 (S536) (catalog no.: 3033, 1:1000 dilution) were obtained from Cell Signaling Technology (Danvers, MA, USA). SOD1 (catalog no.: ab13498, 1:2000 dilution), Cyclin D1 (catalog no.: ab134175, 1:2000 dilution), and p21 (catalog no.: ab109520, 1:1000 dilution) were obtained from Abcam (Cambridge, England, UK). GPX4 (catalog no.: ARG41400, 1:2000 dilution) was obtained from Arigo biolaboratories. Secondary KPL Peroxidase-labeled antibodies were used against Goat anti-mouse IgG (5220-0341, 074-1806, 1:3000 dilution) and Goat anti-rabbit IgG (5220-0336, 074-1506, 1:5000 dilution) (SeraCare, Milford, MA, USA). Immunoreactive proteins were detected using ECL™ Western Blotting Detection Reagent and Amersham Hyperfilm™ ECL (GE Healthcare, Chicago, IL, USA).

### 2.6. Public Transcriptome Reanalysis of GSE193817

To provide orthogonal transcriptomic context for the DFO-induced phenotype observed in HEsSMCs, we reanalyzed the public RNA-seq dataset GSE193817, which profiles human aortic vascular smooth muscle cells isolated from ascending aortas of heart-transplant donors and cultured under quiescent or proliferative conditions [24]. Processed expression matrices for quiescent and proliferative cells were downloaded from the Gene Expression Omnibus (GEO). Ensembl gene identifiers were mapped to HGNC gene symbols using org.Hs.eg.db, and duplicated gene symbols were collapsed by averaging expression values.

The GEO record describes gene expression profiling of smooth muscle cells from approximately 150 donors. The processed matrices used here contained 284 samples, consisting of 139 quiescent and 145 proliferative samples from 148 identifiable donor IDs. For global transcriptomic visualization, principal component analysis (PCA) was performed using the top 2000 most variable genes across all available processed samples (n = 284). For donor-matched analyses, only donors with both quiescent and proliferative samples were retained (n = 136 matched donor pairs). Expression values were analyzed as log_2_(normalized expression + 1).

### 2.7. Differential Expression and Hallmark Pathway Analysis

Differential expressions between quiescent and proliferative smooth muscle cells was estimated using a donor-paired limma model [25]. The moderated t statistic from the quiescent-versus-proliferative contrast was used to rank genes for gene set enrichment analysis. Hallmark pathway enrichment was performed with fgsea using the MSigDB 2025.1.Hs Hallmark collection [26]. Representative Hallmark pathways shown in the figure include E2F targets, G2M checkpoint, hypoxia, myogenesis, angiogenesis, and p53 pathway.

### 2.8. HEsSMC-Related Marker Analysis

Genes selected to contextualize the DFO-HEsSMC phenotype were grouped into six functional categories. The cell-cycle category included CDKN1A, CDKN2A, CCND1, CCNB1, and MKI67. The hypoxia/metabolism category included HIF1A, EPAS1, EGLN3, VEGFA, SLC2A1, PGK1, and IGFBP3. The signaling category included AKT1, MTOR, EGFR, RELA, MAPK1, MAPK3, and SIRT1. The stress/autophagy category included SQSTM1, MAP1LC3B, GPX4, HMOX1, and SOD1. The AngII receptor category included AGTR1 and AGTR2. The smooth muscle cell state category included ACTA2, TAGLN, CNN1, MYH11, DES, COL1A1, COL3A1, VIM, and FN1.

Marker-level expression changes were summarized by log_2_FC (Quiescent—Proliferative) and further visualized as donor-paired expression plots. Statistical significance for paired marker comparisons was evaluated using paired Wilcoxon signed-rank tests.

### 2.9. Curated AngII-Axis Module Analysis

To examine whether AngII-associated signaling might intersect with the DFO phenotype, five curated AngII-axis modules were assembled a priori. The AngII upstream RAAS module comprised AGT, REN, ACE, ACE2, AGTR1, AGTR2, MAS1, MME, ANPEP, LNPEP, CTSA, CMA1, and ATP6AP2. The Immediate early NFKB/AP-1 module comprised JUN, JUNB, JUND, FOS, FOSB, FOSL1, EGR1, EGR2, EGR3, NFKB1, NFKB2, RELA, RELB, NFKBIA, NFKBIZ, TNFAIP3, DUSP1, DUSP5, and ATF3. The PI3K-AKT-MAPK module comprised EGFR, SRC, PIK3CA, PIK3CB, PIK3R1, AKT1, AKT2, MAPK1, MAPK3, MAP2K1, MAP2K2, RAF1, BRAF, ELK1, and CREB1. The PLC-Ca^2+^-contractile module comprised PLCB1, PLCB3, PLCG1, PLCG2, ITPR1, ITPR2, ITPR3, CACNA1C, CALM1, CALM2, CALM3, RHOA, ROCK1, ROCK2, MYLK, MYL9, ACTA2, TAGLN, CNN1, and MYH11. The Remodeling/vasoactive output module comprised EDN1, NOS3, VCAM1, ICAM1, SERPINE1, COL1A1, COL3A1, FN1, TGFB1, CTGF, CYR61, MMP2, and MMP9.

Module enrichment was assessed by fgsea using the same donor-paired limma ranking. Sample-level module scores were calculated as the averaged gene-wise z score across genes detected in each module and compared between quiescent and proliferative samples using paired Wilcoxon signed-rank tests. Core AngII-axis genes, defined as the union of detected genes from the five curated modules, were visualized by ComplexHeatmap, with samples ordered by state and donor, rows grouped by module, top annotation indicating proliferative or quiescent state, and right-side bar annotations indicating gene-level log_2_FC (Quiescent—Proliferative).

### 2.10. Statistics

Bar graphs display individual data points that correspond to biological replicates. In the case of line graphs, the number of biological replicates is indicated in the respective figure legends. Data are expressed as the mean ± standard error of the mean (SEM). Statistical analyses were conducted using GraphPad Prism (version 9, GraphPad Software, San Diego, CA, USA). Depending on the experimental design, either a one-way ANOVA followed by Tukey’s post hoc test, a two-way ANOVA followed by Dunnett’s multiple-comparisons test, or a two-tailed Student’s t-test was employed. Error bars represent the SEM. Statistical significance is defined as follows: * *p* < 0.05, ** *p* < 0.01, and *** *p* < 0.001.

## 3. Results

### 3.1. The Cellular Effects on Cytotoxicity, Cell Cycle Profile, and Reactive Oxygen Species by Deferoxamine and Dimethyloxalylglycine in Human Esophageal Smooth Muscle Cells

**Figure 1:**
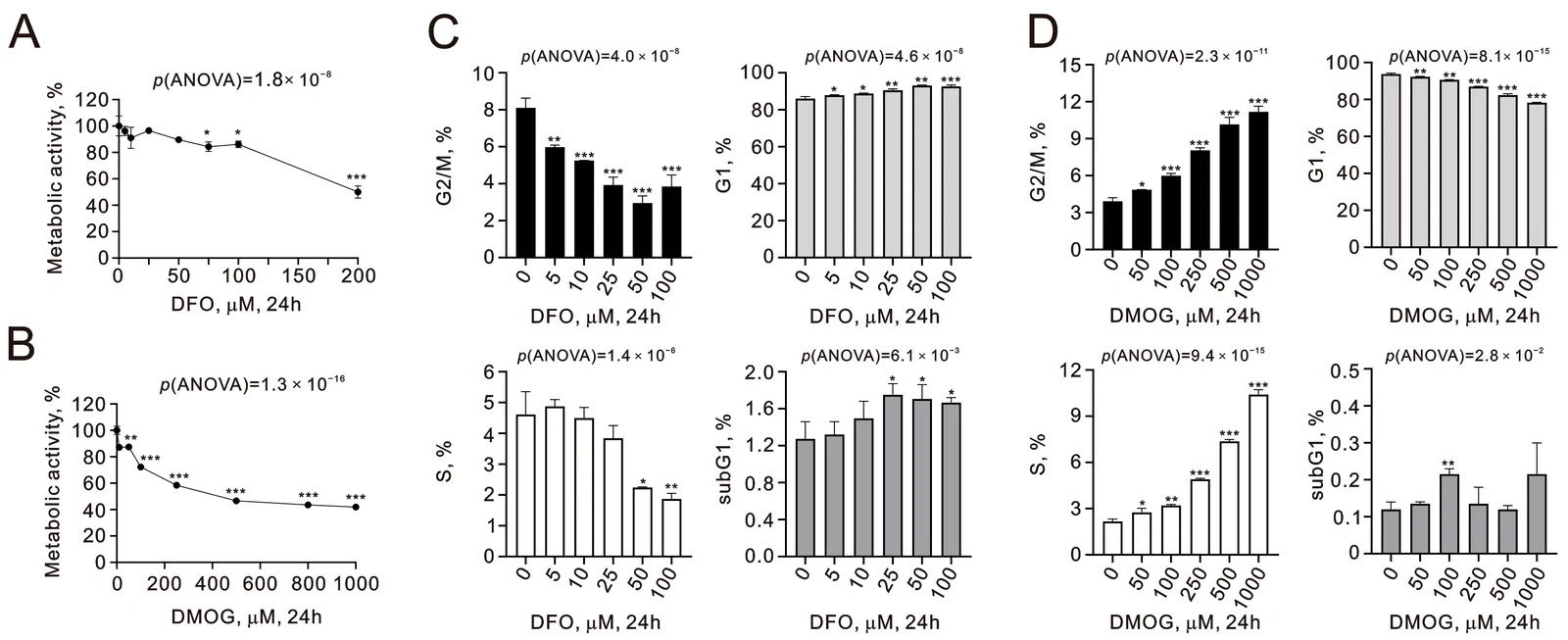
**Effects of DFO and DMOG on metabolic activity and cell cycle profile in HEsSMCs.** HEsSMCs were treated with the specified concentrations of DFO and DMOG for 24 h. (**A**,**B**) Cell metabolic activity was assessed using the MTT assay. (**C**,**D**) Cell cycle profiles were analyzed through flow cytometry using PI staining. The bars in panels (**A**–**D** ) represent the mean ± SD from three independent experiments. Statistical significance was evaluated using Student’s t-test, with comparisons made to the vehicle control; * *p* < 0.05, ** *p* < 0.01, and *** *p* < 0.001.

DFO is commonly used in clinical settings to treat iron overload. In laboratory conditions, it induces a hypoxic response by chelating the essential metal core from enzymes [16]. As a result, HIF stabilization with DFO may take slightly longer to reach peak levels. In contrast, DMOG mimics the structure of α-KG and occupies the active site of the PHD enzyme, competitively inhibiting the binding of the true α-KG. This makes DMOG a more potent and rapid stabilizer of HIF-1α. In this study, we investigated the functional roles of the two chemical hypoxia mimics, DFO and DMOG, in human esophageal smooth muscle cells (HEsSMCs). Using the MTT assay, we found that metabolic activity was reduced to 50% by 200 μM DFO and 400 μM DMOG in HEsSMCs (Figure 1A,B). We further analyzed the impact of DFO and DMOG on the cell cycle profile of HEsSMCs. The data indicated an increasing trend in the sub-G1 phase, with notable differences in the modulation of other phases between DFO and DMOG (Figure 1C,D). DFO increased the proportion of cells in the G1 phase while simultaneously decreasing the levels in the S and G2/M phases. Conversely, DMOG reduced the proportion of cells in the G1 phase, resulting in elevated levels in the S and G2/M phases.

**Figure 2:**
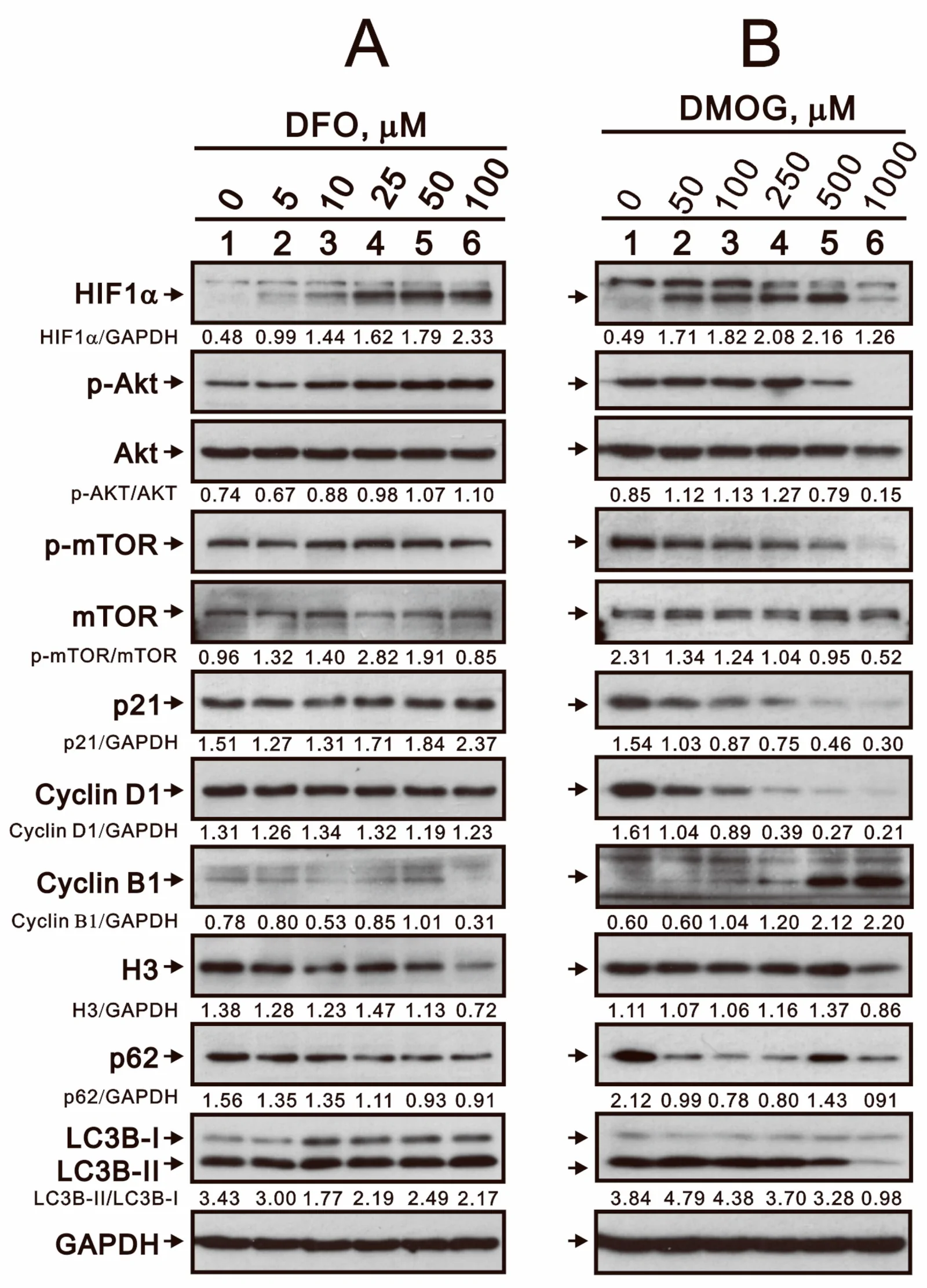
Effects of DFO and DMOG on target proteins expression in HEsSMCs. HEsSMCs were treated with the specified concentrations of (**A**) DFO and (**B**) DMOG for 24 h. Following treatment, cell lysates were analyzed using Western blotting, with GAPDH serving as the loading control. The protein bands in panels (**A**,**B**) were quantified by pixel density scanning and evaluated using ImageJ, version 1.44a (http://imagej.nih.gov/ij/) (accessed on 1 May 2026). The ratios of phosphorylated protein to total protein, protein to GAPDH, and LC3B II to LC3B I are presented.

The effects of DFO and DMOG on HIF-1α stabilization were confirmed through Western blot analysis (Figure 2A,B). In DFO-treated HEsSMCs, we observed a dose-dependent increase in the levels of p-AKT/AKT, p-mTOR/mTOR, and LC3BII/I, alongside a decrease in histone H3 and p62 proteins (Figure 2A). In contrast, while DMOG increased the expression level of cyclin B1, it suppressed the levels of other tested proteins, including p-AKT/AKT, p-mTOR/mTOR, p21, cyclin D1, histone H3, p62, and LC3BII/I in a dose-dependent manner (Figure 2B).

**Figure 3:**
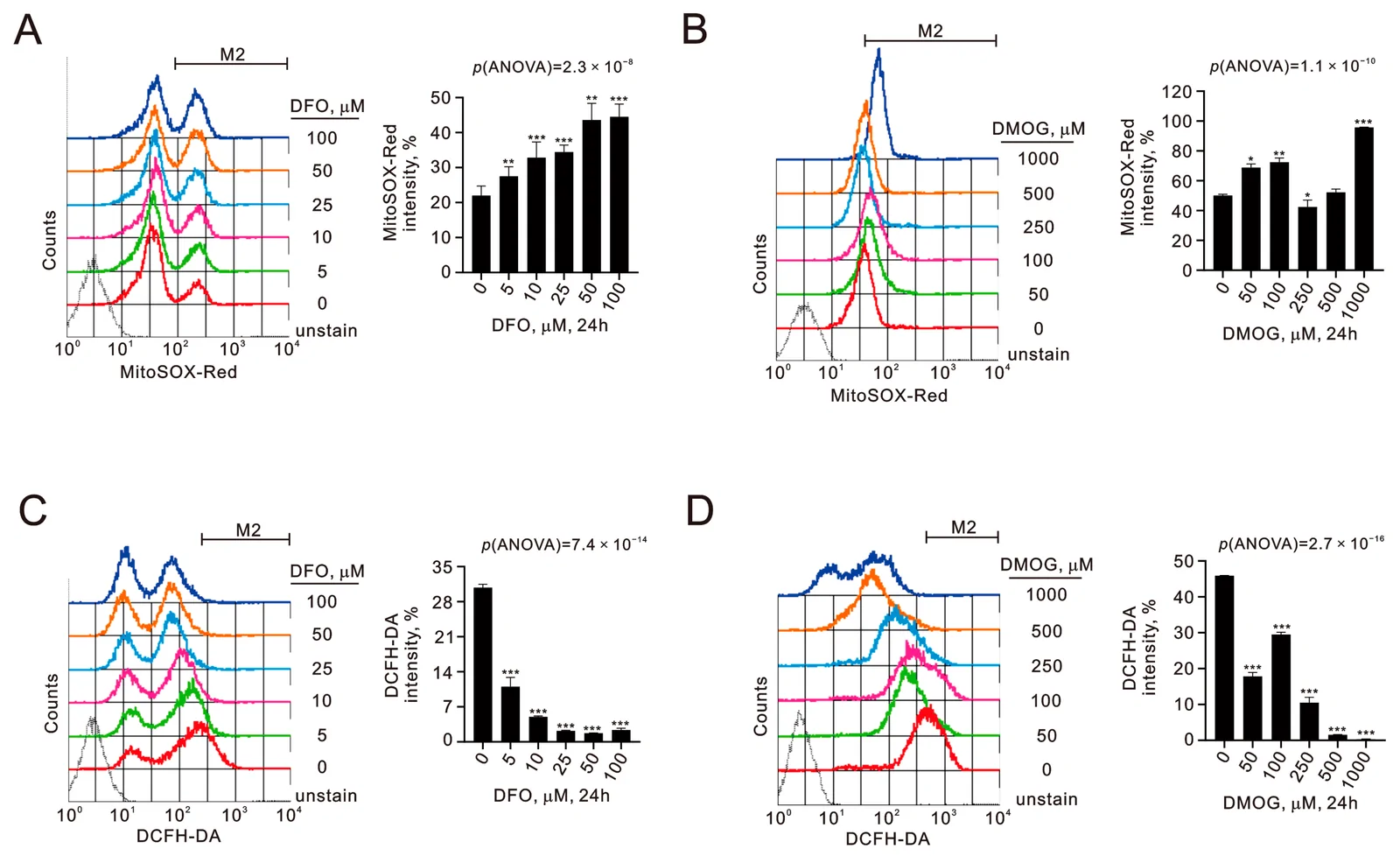
Effects of DFO and DMOG on cytosol and mitochondrial ROS in HEsSMCs. HEsSMCs were treated with the specified concentrations of DFO and DMOG for 24 h. (**A**,**B**) Cytosolic ROS were measured using flow cytometry with DCFH-DA staining. (**C**,**D**) Mitochondrial ROS levels were assessed using flow cytometry with MitoSOX-Red staining. The fluorescence intensity of DCFH-DA and MitoSOX-Red in the vehicle group was used as the baseline for M2 gating. The bars in panels (**A**–**D**) represent the mean ± SD from three independent experiments. Statistical significance was assessed using Student’s *t*-tests, comparing to the vehicle control; * *p* < 0.05, ** *p* < 0.01, and *** *p* < 0.001.

Smooth muscle cells possess a high density of mitochondria to support their contraction [1]. DFO is particularly effective at removing iron from iron-sulfur (Fe-S) clusters within the mitochondrial electron transport chain [16]. When iron is depleted from Complexes I and III, the flow of electrons becomes disrupted. These unpaired electrons can then react with molecular oxygen to generate superoxide, which is subsequently converted to hydrogen peroxide, initiating a chain reaction of lipid peroxidation in both mitochondrial and plasma membranes. Given the implications of DFO and DMOG in ROS generation, we needed to investigate their effects in HEsSMCs. We employed MitoSox-red and DCFH-DA dyes to assess the impact of DFO and DMOG on mitochondrial and cytosolic ROS levels, respectively (Figure 3). Our results demonstrated that both DFO and DMOG increased the levels of mitochondrial ROS (Figure 3A,B) while reducing cytosolic ROS levels (Figure 3C,D).

### 3.2. The Effects of DFO on Cellular Apoptosis, Proliferation, Hypoxia, ROS, Lipid Peroxidation and Mitochondrial Membrane Potential in HEsSMCs

**Figure 4:**
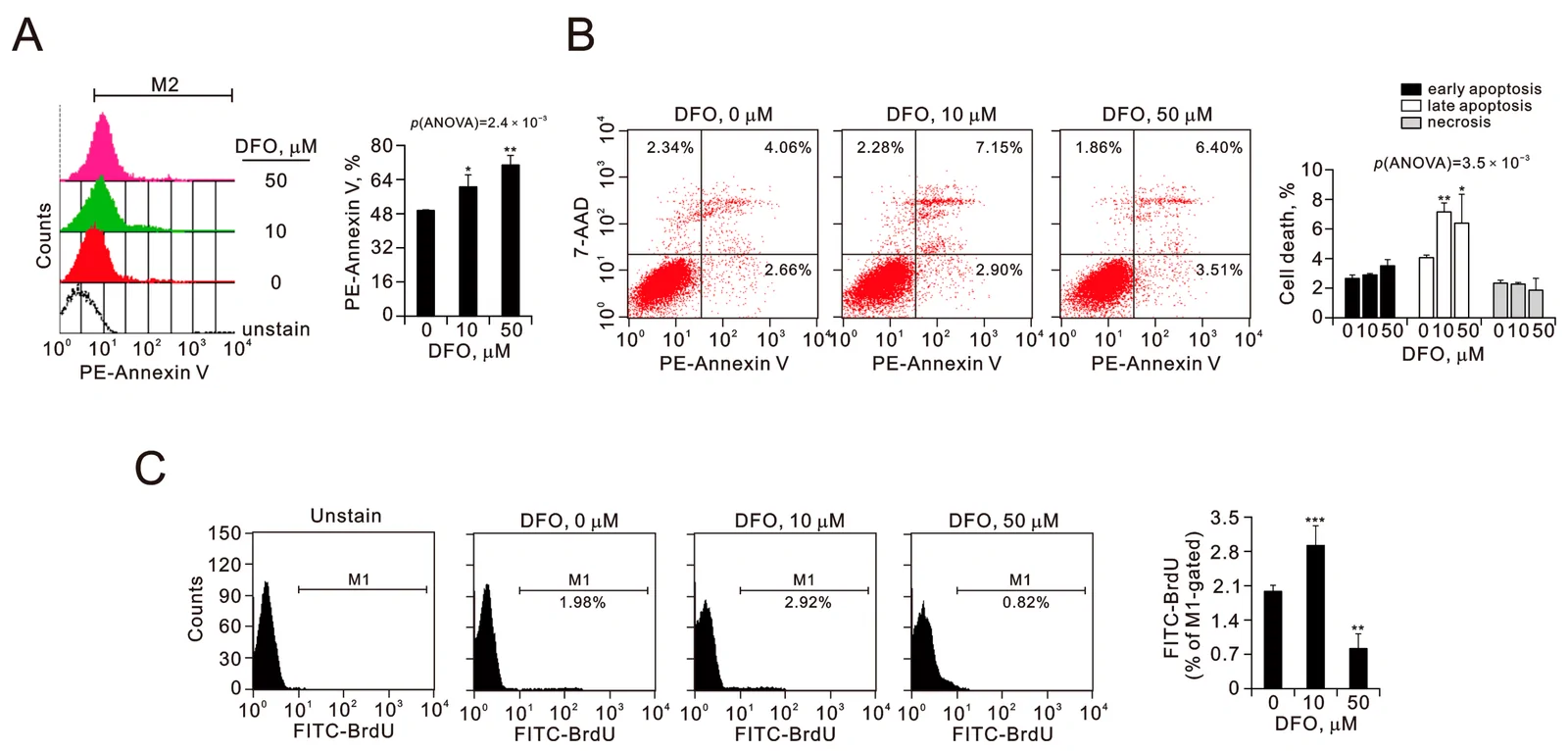
Effects of DFO on cell apoptosis and proliferation in HEsSMCs. HEsSMCs were treated with the specified concentrations of DFO for 24 h. (**A**) Cellular apoptosis was assessed using PE-Annexin V staining combined with 7-AAD. The fluorescence intensity of PE-Annexin V in the vehicle group served as the baseline for M2 gating. (**B**) Early apoptotic cells were identified as PE-Annexin V positive and 7-AAD negative, while late apoptotic cells were both PE-Annexin V positive and 7-AAD positive. Necrotic cells were characterized as PE-Annexin V-negative and 7-AAD-positive. (**C**) Cell proliferation was evaluated through flow cytometry by incorporating FITC-BrdU. The bars in panels (**A**–**C**) represent the mean ± SD from three independent experiments. Statistical significance was determined using Student’s *t*-tests, with comparisons made to the vehicle control; * *p* < 0.05, ** *p* < 0.01, and *** *p* < 0.001.

Compared to the laboratory drug DMOG, DFO is marketed under the brand name Desferal^®^ and is specifically used to treat conditions such as iron overload, hemochromatosis (whether due to multiple blood transfusions or genetic predispositions), and aluminum toxicity in dialysis patients [27]. In our study, we noted a dose-dependent effect of DFO—unlike DMOG—on mitochondrial and cytosolic ROS levels in HEsSMCs. Therefore, we focused on DFO to investigate its effects on cellular apoptosis and proliferation (Figure 4). Our flow cytometry analysis using annexin-V/7-AAD revealed that DFO induced cellular apoptosis, including late-stage apoptotic cells (Figure 4A). In addition, the BrdU flow cytometry results indicated that DFO stimulated cellular proliferation at a concentration of 10 μM but inhibited proliferation at 50 μM (Figure 4B).

**Figure 5:**
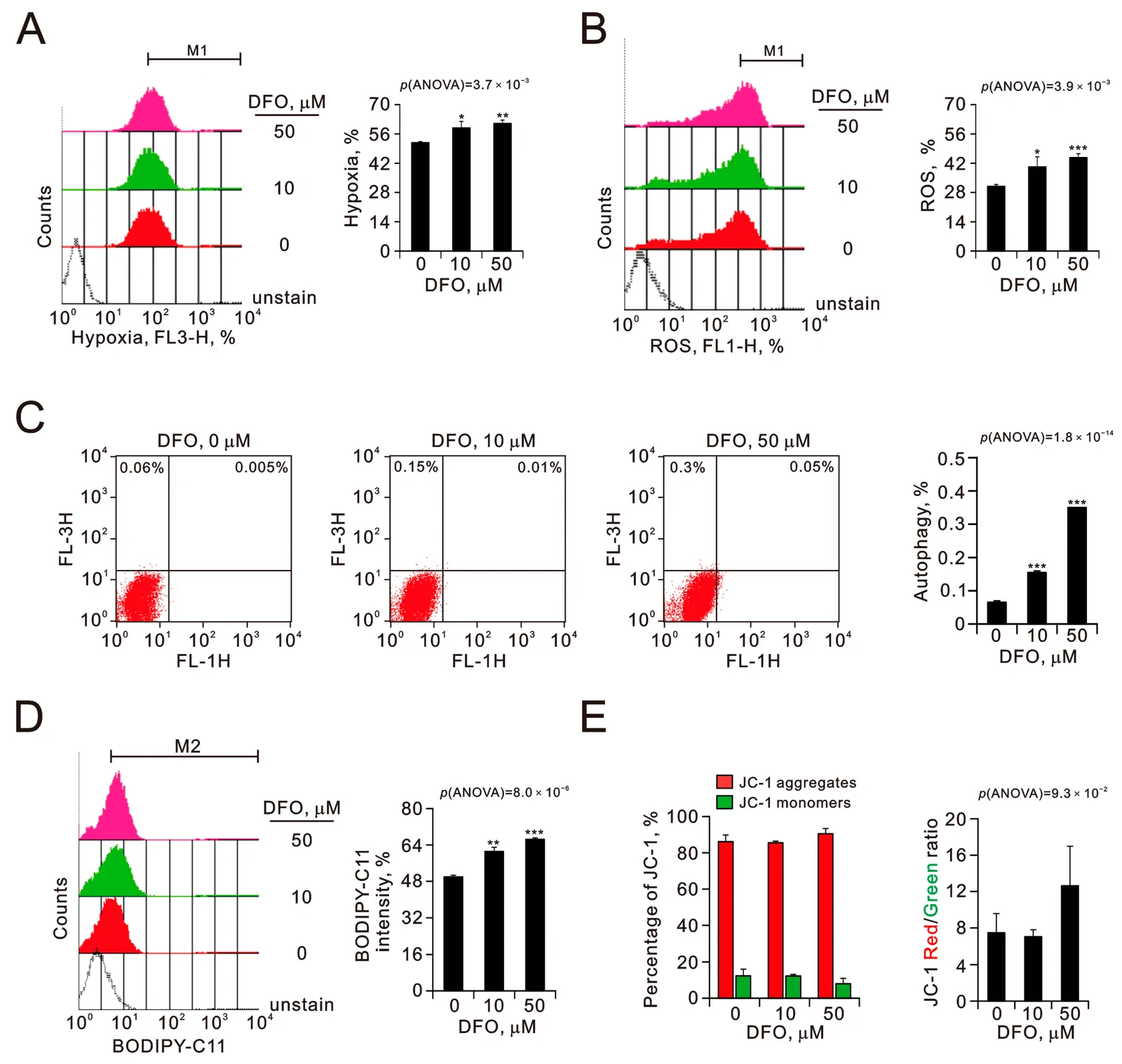
Effects of DFO on cell hypoxia-oxidative status, autophagy, lipid peroxidation, and mitochondrial membrane potential in HEsSMCs. HEsSMCs were treated with the specified concentrations of DFO for 24 h. (**A**) To evaluate the level of cell hypoxia, flow cytometry was performed using Hypoxia Red dye staining, and the percentage of hypoxic cells (as identified by the M1-gated population) was quantified. (**B**) The level of ROS in the cells was assessed by staining with an oxidative stress detection dye, followed by flow cytometric analysis to quantify the percentage of ROS-positive cells (also in the M1-gated population). (**C**) Autophagic cells were identified using acridine orange staining, with flow cytometric analysis being used to assess the intensity of red fluorescence (y-axis, FL3-H), which indicates the degree of acidity and the volume of acidic vesicular organelles, including autophagic vacuoles. (**D**) Lipid peroxidation was measured through flow cytometry with BODIPY-C11 staining, and the resulting fluorescence intensity was plotted. (**E**) The mitochondrial membrane potential was evaluated using flow cytometry with JC-1 dye staining, and the percentages of red and green fluorescence intensities were plotted. The ratios of red to green fluorescence were also calculated and depicted. Bars in panels (**A**–**E**) represent the mean ± SD from three independent experiments. Statistical significance was analyzed using Student’s *t*-tests, comparing against the vehicle; * *p* < 0.05, ** *p* < 0.01, and *** *p* < 0.001.

We further applied flow cytometry analyses to assess hypoxia and ROS levels using the ROS-ID Hypoxia/Oxidative Stress Detection Kit, autophagy via acridine orange (AO), lipid peroxidation using BODIPY-C11, and mitochondrial membrane potential with JC-1 across various concentrations of DFO in HEsSMCs (Figure 5). Our findings showed that DFO increased the fluorescent intensity of hypoxia, ROS, autophagy (AO), and lipid peroxidation (BODIPY-C11) in a dose-dependent manner. Notably, the JC-1 red/green ratio also increased with 50 μM DFO treatment.

### 3.3. The Rescue Effect of Angiotensin II on DFO Is Involved in Cellular Stresses of HEsSMCs

The clinical application of AngII has undergone significant changes in recent years [21,28,29]. Once primarily considered a physiological hormone and a target for ACE inhibitors, AngII is now recognized as a direct therapeutic intervention in critical care settings. In 2017, AngII was FDA approved for treating hypotension in adults experiencing septic or other distributive shock. AngII exerts its effects by binding to the AT1R, which activates the production of ROS through NADPH oxidase, subsequently interfering with the activity of PHDs [30]. This generation of ROS or alteration of the redox state prevents the degradation of HIF-1α, allowing its accumulation even in the presence of oxygen [22,23]. Recent research has shown that the human distal esophagus expresses a localized renin–angiotensin system (RAS) [31,32]. This implies that esophageal cells are not only responsive to systemic AngII but may also be influenced by AngII produced locally. Beyond the classical pathway, AngII modulates NF-κB activity through the non-classical Ras/MEK/ERK pathway [33], resulting in a more pronounced inflammatory response in muscle and vascular tissues.

**Figure 6:**
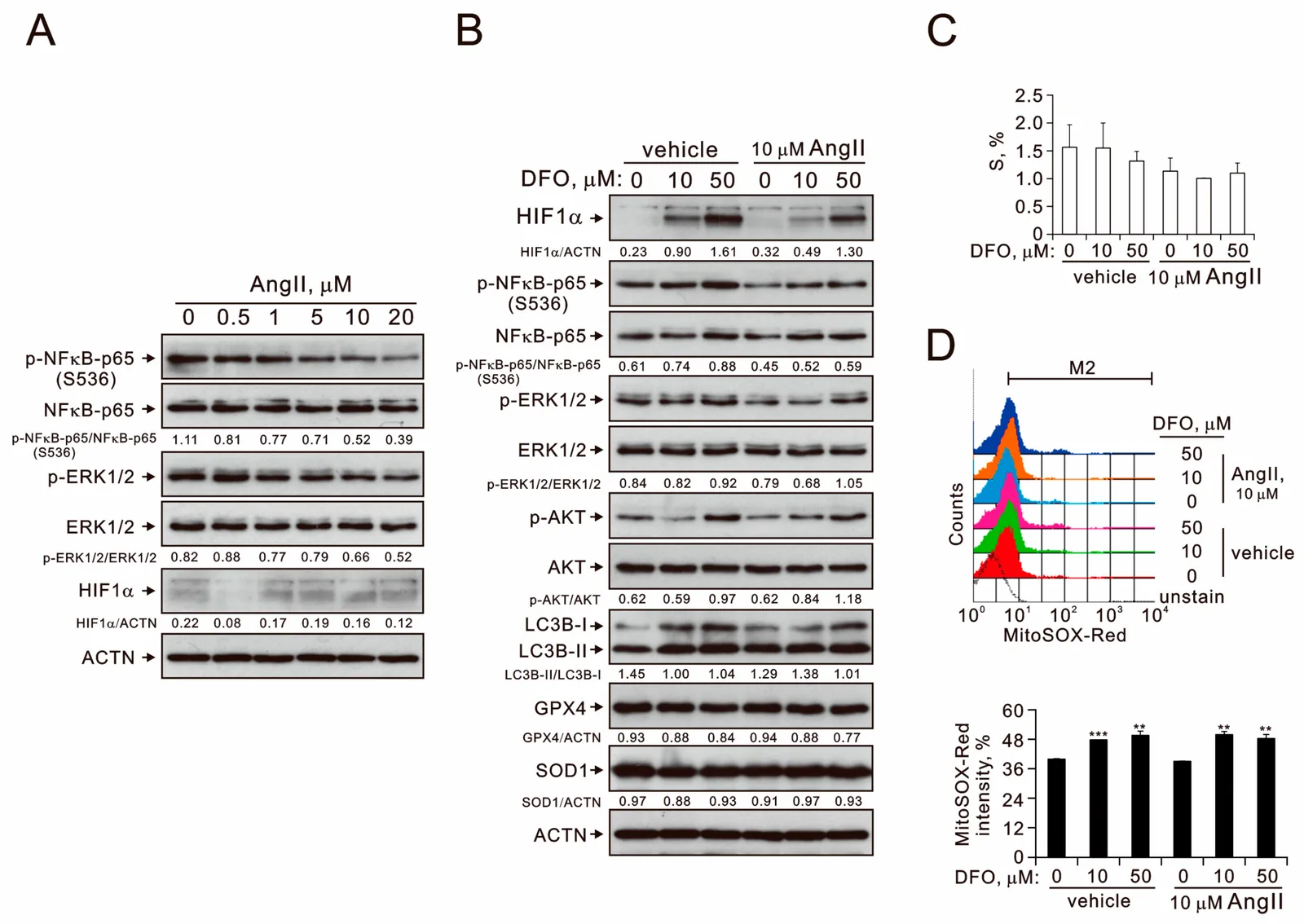
Effects of AngII and AngII combined with DFO on target proteins expression, S-phase, and mitochondrial ROS in HEsSMCs. HEsSMCs were treated with the specified concentrations (0, 0.5, 1, 5, 10, and 20 μM) of Ang II for 24 h. (**A**) Cell lysates were analyzed via Western blot, with ACTN serving as the protein loading control. HEsSMC cells were treated with the specified concentrations (0, 10, and 50 μM) of DFO, either alone or in combination with 10 μM Ang II, for a duration of 24 h. (**B**) These cell lysates were also subjected to Western blot analysis, using ACTN as the loading control. The protein bands from both (**A**,**B**) were quantified by pixel density scanning and evaluated with ImageJ version 1.44a (http://imagej.nih.gov/ij/) (accessed on 1 May 2026). The ratios of phosphorylated protein to total protein or protein to ACTN were recorded. (**C**,**D**) HEsSMC cells were treated with the specified concentrations (0, 10, and 50 μM) of DFO, either alone or in combination with 10 μM Ang II, for a duration of 24 h. (**C**) Cell proliferation was assessed using flow cytometric analysis with propidium iodide (PI) staining. (**D**) Mitochondrial reactive oxygen species (ROS) levels were measured using flow cytometry with MitoSOX-Red staining, with the fluorescence intensity of the vehicle used as a baseline for M2 gating. The bars in panel (**D**) represent the mean ± SD of three independent experiments. Statistical significance was determined using Student’s *t*-tests compared to the vehicle; ** *p* < 0.01 and *** *p* < 0.001.

Based on the properties and clinical applications of AngII, we aimed to investigate whether AngII could alleviate DFO-induced hypoxic stress in HEsMCs through Western blotting and flow cytometry analysis. Our Western blot analysis indicated that AngII did not affect the levels of NF-κB, ERK, HIF-1α, or the internal control α-actinin (ACTN). However, it did suppress the expression levels of p-NF-κB and p-ERK in a dose-dependent manner (Figure 6A). Notably, the combination of 10 µM AngII significantly attenuated the inductive effects of DFO on HIF-1α, p-NF-κB, and p-ERK (Figure 6B). Furthermore, our flow cytometry data demonstrated that 10 µM DFO was unable to rescue the suppression of S-phase induced by DFO (Figure 6C), nor did it prevent the DFO-induced generation of mitochondrial ROS in HEsSMCs (Figure 6D).

### 3.4. Public Smooth Muscle Transcriptomes Support a Quiescent, Anti-Proliferative Reference State That Overlaps with Key Components of the DFO-HEsSMC Phenotype

**Figure 7:**
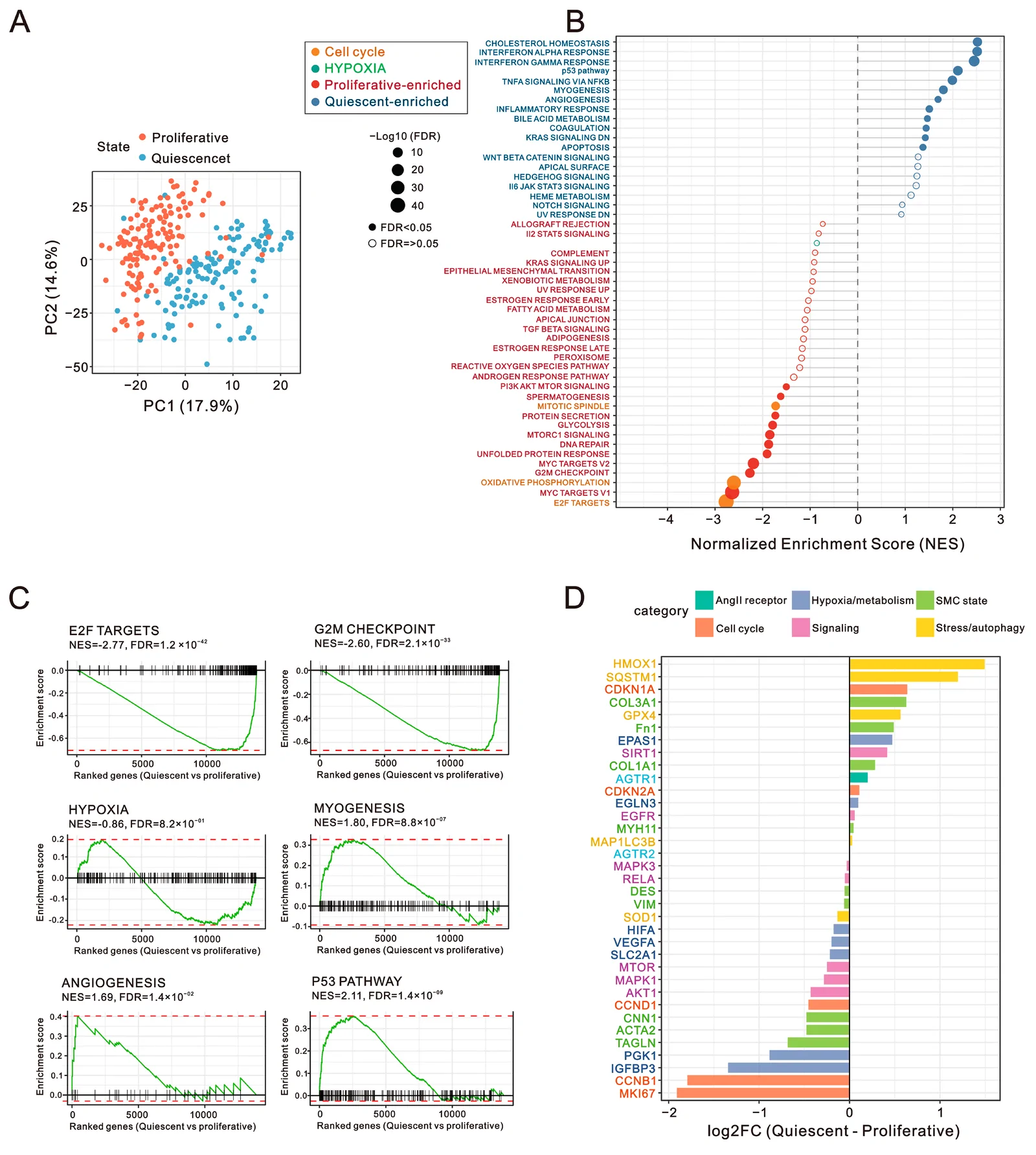
Public transcriptomic reanalysis of GSE193817 defines a quiescent, anti-proliferative smooth muscle reference state. (**A**) Principal component analysis of the top 2000 most variable genes across all processed smooth muscle cell samples (n = 284; 139 quiescent and 145 proliferative samples). Each point represents one sample. (**B**) Hallmark gene set enrichment analysis (GSEA) of all 50 MSigDB Hallmark pathways using genes ranked by the donor-paired limma moderated t-statistic from 136 matched donor pairs. Positive normalized enrichment scores (NES) indicate enrichment in quiescent smooth muscle cells, whereas negative NES values indicate enrichment in proliferative smooth muscle cells. (**C**) Representative GSEA enrichment plots for selected pathways, including E2F targets, G2M checkpoint, hypoxia, myogenesis, angiogenesis, and p53 pathway. (**D**) HEsSMC-related marker summary shown as log_2_FC (Quiescent—Proliferative), with positive values indicating higher mean expression in quiescent smooth muscle cells.

Reanalysis of the GSE193817 dataset revealed a clear distinction between quiescent and proliferative smooth muscle cells as demonstrated by PCA (Figure 7A). This finding supports the use of this dataset as a reliable reference for characterizing smooth muscle cell states. Gene Set Enrichment Analysis (GSEA) across all 50 Hallmark pathways (Figure 7B) further highlighted a significant depletion of proliferative programs in quiescent cells, with marked negative enrichment observed in E2F targets (NES = −2.77, FDR = 1.2 × 10^−42^) and the G2M checkpoint (NES = −2.60, FDR = 2.1 × 10^−33^). Representative enrichment plots (Figure 7C) corroborated the depletion of E2F targets and the G2M checkpoint, while the canonical hypoxia Hallmark signature showed no significant enrichment (NES = −0.86, FDR = 8.2 × 10^−1^). These results suggest that the GSE193817 dataset primarily captures the transition between quiescent and proliferative smooth muscle states rather than a direct hypoxic response. Nonetheless, several pathways related to tissue remodeling and stress adaptation, including myogenesis, angiogenesis, and the p53 pathway, were relatively enriched in quiescent cells.

At the gene level, the summary of HEsSMC-related markers (Figure 7D) demonstrated that several DFO-relevant markers aligned with the quiescent reference state. Positive markers identified in quiescent cells included HMOX1, SQSTM1, CDKN1A, COL3A1, GPX4, FN1, EPAS1, and SIRT1. In contrast, proliferative cells exhibited relatively higher expression levels of MKI67, CCNB1, IGFBP3, PGK1, TAGLN, ACTA2, CNN1, CCND1, AKT1, MAPK1, and MTOR. These findings support the interpretation that the DFO phenotype observed in HEsSMCs is more indicative of an anti-proliferative and stress-remodeling state, rather than a uniform activation of canonical hypoxia signaling.

### 3.5. Donor-Paired Marker Analyses Confirm Coordinated Differences in Cell Cycle, Signaling, Hypoxia-Associated Genes, Stress Pathways, and Smooth Muscle State Markers

**Figure 8:**
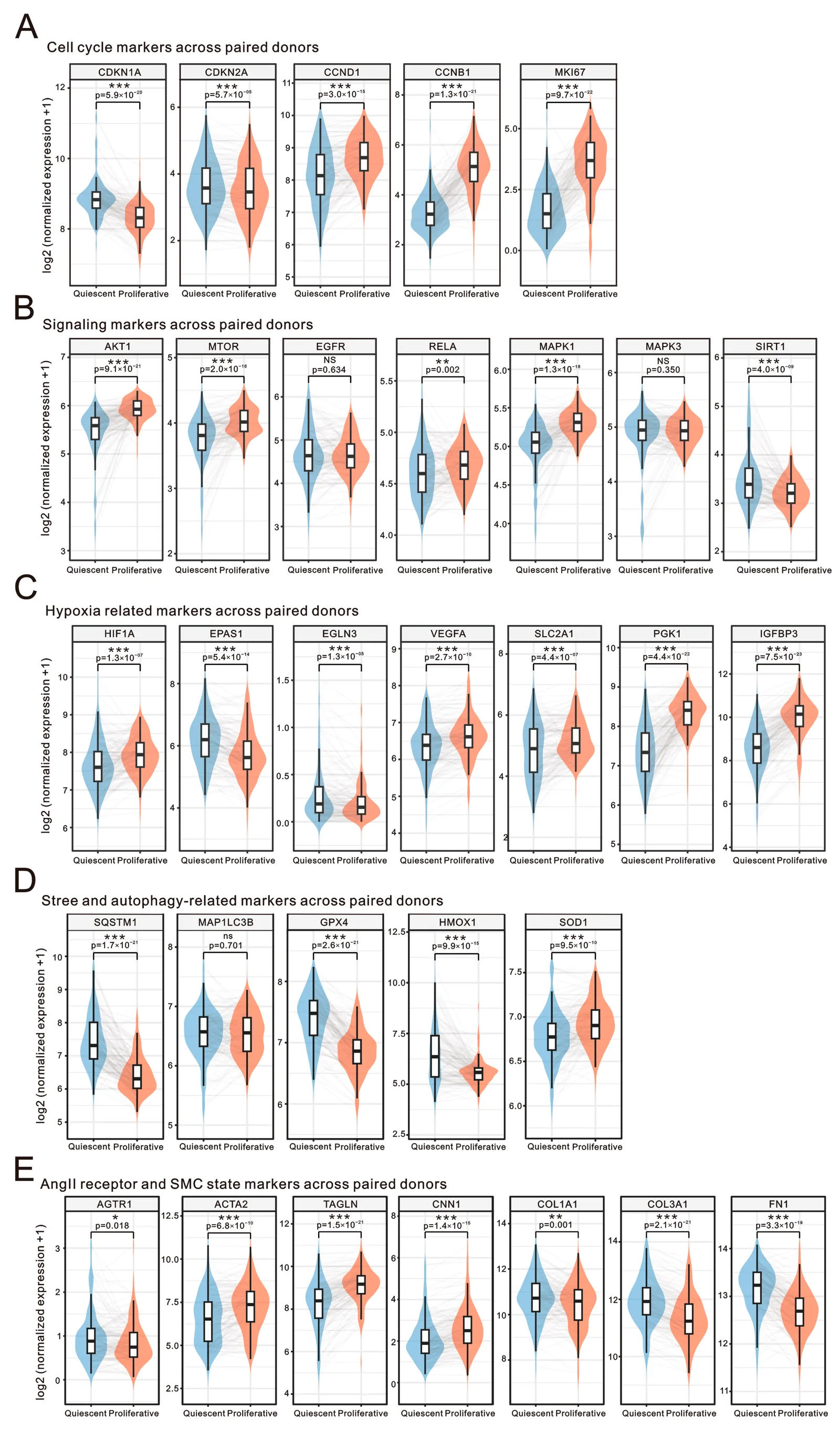
Donor-paired expression analysis of HEsSMC-related marker groups in GSE193817. (**A**–**E**) Donor-matched quiescent and proliferative smooth muscle cell samples from GSE193817 (n = 136 matched donor pairs) were compared for HEsSMC-related marker expression. Expression values are shown as log_2_(normalized expression + 1), and lines connect paired samples from the same donor. *p* values were calculated using paired Wilcoxon signed-rank tests. Marker groups include cell-cycle markers (**A**), signaling-related markers (**B**), hypoxia/metabolism-related markers (**C**), stress/autophagy-related markers (**D**), and AngII receptor and smooth muscle-state markers (**E**). Statistical significance was analyzed using Student’s *t*-tests, comparing against the vehicle; * *p* < 0.05, ** *p* < 0.01, and *** *p* < 0.001.

Paired donor analysis revealed a pronounced anti-proliferative signature in quiescent smooth muscle cells. In the cell-cycle panel (Figure 8A), we observed increases in CDKN1A and CDKN2A, while CCND1, CCNB1, and MKI67 were reduced, indicating cell-cycle suppression. In the signaling panel (Figure 8B), the expression levels of AKT1, MTOR, and MAPK1 were significantly lower in quiescent cells, while SIRT1 was elevated. Hypoxia-associated genes displayed a mixed but informative pattern (Figure 8C). HIF1A, EPAS1, EGLN3, VEGFA, and SLC2A1 were relatively higher in quiescent cells, whereas PGK1 and IGFBP3 showed increased levels in proliferative cells.

Stress and autophagy-related markers (Figure 8D), including SQSTM1, GPX4, HMOX1, and SOD1, were elevated in quiescent cells, while the levels of MAP1LC3B did not differ significantly. In the panel assessing AngII receptors and smooth muscle state markers (Figure 8E), AGTR1 exhibited a modest but significant increase in quiescent cells, whereas ACTA2, TAGLN, and CNN1 were lower. Conversely, matrix-associated markers such as COL1A1, COL3A1, and FN1 were elevated in quiescent cells, suggesting a remodeling-associated phenotype rather than a contractile one.

### 3.6. AngII-Associated Signaling Is Distributed Across Distinct Upstream and Downstream Modules Rather than Supporting a Single Rescue-like Axis

**Figure 9:**
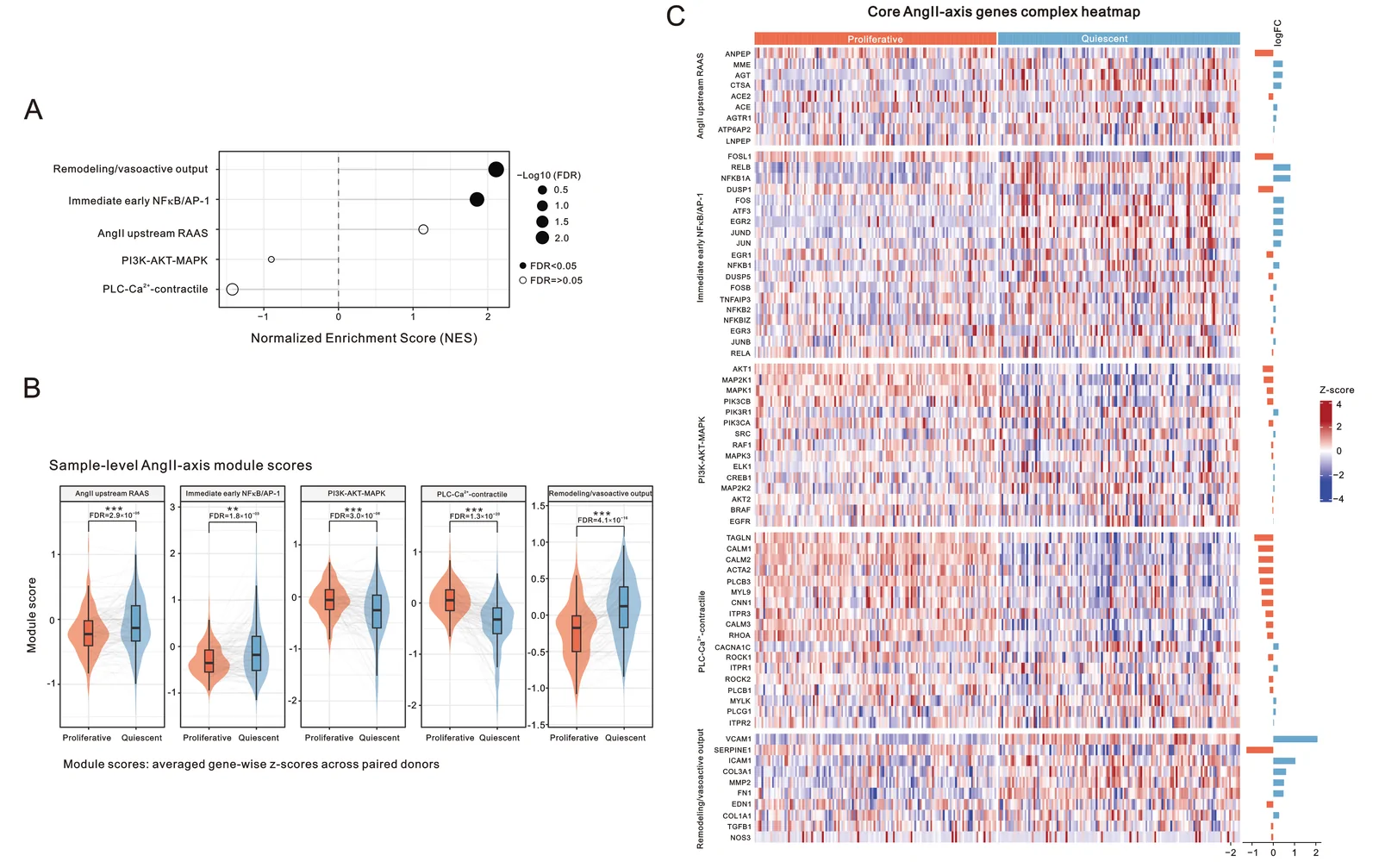
Curated AngII-axis module analysis in GSE193817. (**A**) Gene set enrichment analysis of five curated AngII-axis modules using the donor-paired limma ranked gene list from quiescent and proliferative smooth muscle cells. The predefined modules include AngII upstream RAAS, Immediate early NFKB/AP-1, PI3K-AKT-MAPK, PLC-Ca^2+^-contractile, and Remodeling/vasoactive output. (**B**) Sample-level module scores across 136 matched donor pairs. Scores were calculated as the average gene-wise z score across detected genes in each module and compared between paired quiescent and proliferative samples using paired Wilcoxon signed-rank tests. (**C**) ComplexHeatmap of detected core AngII-axis genes. Columns are ordered by sample state, the top annotation indicates proliferative or quiescent status, rows are grouped by curated module membership, heatmap values represent per-gene z scores, and right-side bar annotations show gene-level log_2_FC (Quiescent—Proliferative). Statistical significance was determined using Student’s *t*-tests compared to the vehicle; ** *p* < 0.01 and *** *p* < 0.001.

Curated analysis of the AngII axis revealed that AngII-related biology in the GSE193817 dataset is modular rather than uniform. At the pathway level (Figure 9A), modules associated with immediate early NF-κB/AP-1 and remodeling/vasoactive responses were shifted towards the quiescent state. In contrast, modules related to PI3K-AKT-MAPK and PLC-Ca^2+^-contractile activity were more closely associated with the proliferative state. Sample-level module scores (Figure 9B) confirmed this trend across donor-matched pairs. The AngII upstream Renin–Angiotensin–Aldosterone System (RAAS) module exhibited a modest shift towards the quiescent state; however, the overall response associated with AngII did not behave as a singular, coordinated pathway.

Visualization of core AngII-axis genes using a complex heatmap (Figure 9C) further illustrated that RAAS-associated genes and immediate early response genes were intermixed with module-specific directional changes. Notably, many PLC-Ca^2+^-contractile genes were relatively higher in proliferative cells, while several remodeling-associated output genes were more prevalent in quiescent cells. Collectively, these findings suggest that AngII-related signaling may intersect with the DFO phenotype at the level of selective downstream programs, but they do not support a simplistic AngII-rescue model within this reference dataset.

## 4. Discussion

In this study, we employed primary HEsSMCs to investigate the effects of chemical hypoxia mimics, such as DFO and DMOG, and to explore the potential cross-talk between hypoxia and AngII. We integrated the public RNA sequencing dataset GSE193817 to provide an orthogonal transcriptomic context for the DFO-induced phenotypes observed in HEsSMCs. Our results indicated that DFO and DMOG stabilized HIF-1α proteins but did so at different time points. In addition to exhibiting distinct cell cycle profiles, various cellular proteins were affected by DFO and DMOG in divergent trends within HEsSMCs. Notably, DFO induced mitochondrial ROS while suppressing cytosolic ROS, suggesting that multiple targets were not directly related to hypoxic stress. Further flow cytometry data revealed that DFO induced late-stage apoptosis, hypoxia, an overall increase in ROS, autophagy, lipid peroxidation, and mitochondrial membrane depolarization in HEsSMCs. The inactivation of NF-κB induced by AngII also led to the suppression of NF-κB activation mediated by DFO in HEsSMCs. Reanalysis of the GSE193817 dataset demonstrated a clear distinction between quiescent and proliferative smooth muscle cells, indicating that the dataset primarily captures the transition between these states rather than a direct hypoxic response. Curated analysis of the AngII axis revealed that AngII-related biology in GSE193817 is modular rather than uniform. Therefore, while AngII-related signaling may intersect with the DFO phenotype at the level of selective downstream programs, it does not support a simplistic AngII-rescue model within this reference dataset.

The half-life of DFO in humans is approximately 20 to 30 min; however, it supports significant HIF-1α accumulation for 12 to 24 h in culture before its iron-binding capacity becomes saturated or cellular homeostasis adjusts. DMOG quickly undergoes de-esterification to produce monomethyl-oxalylglycine (MOG), which has an in vitro half-life of about 10 to 15 min. MOG is transported into cells via the monocarboxylate transporter 2, where it is further cleaved by intracellular esterases into the active metabolite N-oxalylglycine (NOG). Once inside the cells, the conversion to NOG leads to the functional inhibition of PHDs, which typically maintains elevated HIF-1α levels for an additional 12 to 24 h. Chemical hypoxia differs from atmospheric hypoxia in that it increases the NADH/NAD^+^ ratio and alters mitochondrial ROS levels [3,6,16]. Unlike atmospheric hypoxia, DFO and DMOG act directly on the PHD enzymes, bypassing the mitochondria altogether. While HIF-1 stabilization with DFO may take slightly longer to reach peak levels, DMOG is often recognized as a more potent and rapid stabilizer of HIF-1α. DFO is particularly effective at inhibiting factor inhibiting HIF (FIH) due to FIH’s high sensitivity to iron depletion. Consequently, DFO may yield a higher quality hypoxic response in terms of HIF-1α transcriptional activity, as it effectively shuts down both the degradation of PHD and the inactivation of FIH through iron deprivation. Furthermore, DFO and DMOG exhibited distinct patterns in their effects on cell cycle profiles, including the G1, S, and G2/M phases, as well as differing dose-dependent trends in target proteins such as p-AKT/AKT, p-mTOR/mTOR, p21, cyclin D1, cyclin B1, and LC3B II/I. Our current findings suggest that chemical hypoxia mimetics like DFO and DMOG induce stability of the HIF-1α protein through their unique metabolic pathways, leading to different cellular responses mediated by specialized proteins.

Our current findings indicate that DFO increases the levels of ROS, particularly mitochondrial ROS. This suggests that the ratio of DFO to iron may not be sufficiently high to completely sequester the metal, leading to the formation of intermediates during the oxidation process that can trigger a significant burst of ROS. When DFO binds to Fe^2+^, it rapidly oxidizes it to Fe^3+^. The Fenton reaction occurs when hydrogen peroxide reacts with Fe^2+^ to generate hydroxyl radicals and additional ROS [34]. Conversely, the reduction of Fe^3+^ by hydrogen peroxide regenerates Fe^2+^ and produces hydroperoxyl radicals. HEsSMCs may already have elevated baseline levels of hydrogen peroxide, and the introduction of DFO can accelerate the initial oxidation of iron, resulting in a temporary spike in radical production that initiates a chain reaction of lipid peroxidation. While DFO is commonly used as a potent inhibitor of lipid peroxidation and ferroptosis, our data demonstrates that it also increases levels of lipid peroxidation. This is consistent with the requirement of free iron for catalyzing the production of hydroxyl radicals, indicating that the ratio of DFO to iron is insufficient to fully sequester the metal in HEsSMCs. Co-treatment with mitochondria-targeted antioxidants such as MitoQ or MitoTEMPO alongside DFO will help clarify the mitochondrial sources of lipid peroxidation in HEsSMCs.

AngII primarily exerts its effects through interaction with the AT1R, which activates downstream signaling cascades that significantly increase the production of intracellular ROS [22,23]. Research has shown that the human distal esophagus expresses a localized RAS [31,32]. This surge of ROS results in a localized decrease in intracellular ascorbate, an essential co-factor for PHDs, leading to the stabilization of HIF-1α protein [35]. AngII is known to induce mitochondrial dysfunction, resulting in the generation of mitochondrial ROS, which modulates various responses to AngII, including the development of experimental hypertension [36]. However, our data indicated that AngII had no effect on HIF-1α protein levels or DFO-induced mitochondrial ROS in HEsSMCs. Beyond stabilizing HIF-1α, AngII-induced ROS also promotes the degradation of IκB, thereby activating NF-κB activity, including the phosphorylation of the p65 subunit [33]. Moreover, AngII stimulates cellular proliferation via the MEK/ERK pathway and enhances cytokine production through the IKK/p65 pathway [37,38]. NF-κB serves as a critical mediator of intracellular signaling by AngII under both normal physiological conditions and after vascular injury [39]. NF-κB activation is essential for AngII-dependent proliferation and migration of vascular smooth muscle cells. In contrast, we observed that AngII suppressed the levels of p-p65 and p-ERK1/2 in HEsSMCs. One study suggested that AngII can exert an inhibitory effect on inflammation by downregulating the prolonged activation of ERK and NF-κB [40]. Additionally, the ACE2/Ang-(1-7)/Mas axis has been shown to protect against lung fibrosis by inhibiting the MAPK/NF-κB pathway [41]. Hence, it is an interesting issue to address the context-dependent manner of HEsSMCs for the functional role of AngII, including its working form and target receptor.

Smooth muscle cells exhibit a high degree of phenotypic plasticity, allowing them to transition between a quiescent/contractile state and a proliferative/synthetic state [1]. Chronic hypoxia is a well-established driver of smooth muscle phenotypic modulation, often pushing cells from their quiescent, contractile state into an activated, proliferative phenotype [2,6]. In the context of HEsSMCs, “quiescent” and “hypoxic” refer to entirely distinct cellular states: one represents a physiological baseline phenotype, while the other denotes a condition of microenvironmental stress. Reanalysis of the GSE193817 dataset indicates that the DFO phenotype observed in HEsSMCs aligns more closely with an anti-proliferative and stress-remodeling state than with a uniform activation of canonical hypoxia signaling. Our flow cytometry analysis and Western blotting data support the conclusion that DFO modulates cellular proliferation, induces G1 cell cycle arrest, influences autophagy, and alters the levels of p21 and cyclin D1 proteins. In HEsSMCs, DFO deprives ribonucleotide reductase of iron, rendering the enzyme inactive and resulting in an immediate shortage of deoxyribonucleotide triphosphates. Cellular replication checkpoints promptly detect the absence of the necessary nucleotide building blocks required for progression to the S-phase. Simultaneously, iron deprivation leads to the downregulation of G1 cyclins and cyclin-dependent kinases (CDKs), such as Cyclin D1/CDK4, while cell-cycle inhibitors like p21 and p27 are upregulated, effectively locking the cells permanently in the G1 phase [42]. Consequently, our data suggest that HEsSMCs halt their cell cycle at the G1 phase to conserve energy, activate a hypoxic program via HIF-1α for metabolic adaptation, and initiate autophagy to eliminate damaged organelles and recycle structural materials for survival. Furthermore, the chemical hypoxia induced by DFO may operate through mechanisms distinct from the canonical hypoxia signaling pathway associated with low oxygen tension, highlighting the differences between DFO and DMOG.

In this study, DFO appears to effectively mimic the structural and metabolic stress associated with ischemic tissue [27]. However, there are several limitations to using DFO as a hypoxia mimic under current experimental conditions. Besides not representing true hypoxic status, culture media containing high levels of fetal bovine serum can interfere with the efficacy of DFO, as serum contains transferrin and free iron that can neutralize DFO before it reaches the cells. The chelation of iron influences all iron-dependent enzymatic activities, which not only stabilizes HIF-1α protein but also disrupts the cellular functions of other important proteins. In the current study, DFO-induced mitochondrial oxidative stress, lipid peroxidation, autophagy, cell-cycle arrest, and apoptosis can diminish smooth muscle cell viability and potentially hinder smooth muscle function. Likewise, similar mechanisms involving hypoxia, oxidative stress, and phenotypic modulation of smooth muscle contribute to pulmonary vascular remodeling in pulmonary hypertension and airway smooth muscle remodeling in asthma. However, since the present study was conducted using HEsSMCs and the GSE193817 dataset, these broader implications remain speculative and need validation in tissue-specific smooth muscle cells and appropriate patient-derived transcriptomic and clinical data.

## 5. Conclusions

In this study, we utilized HEsSMCs to investigate the effects of chemical hypoxia mimics, such as DFO and DMOG, while also examining the interplay between hypoxia and AngII. Our findings reveal that both DFO and DMOG stabilize HIF-1α proteins, albeit at different time points, and demonstrate distinct cell cycle profiles and divergent effects on various cellular proteins within HEsSMCs. Notably, flow cytometry analysis showed that DFO triggers late-stage apoptosis, induces hypoxic conditions, increases ROS, and promotes autophagy, lipid peroxidation, and mitochondrial membrane depolarization. Furthermore, the inactivation of NF-κB by AngII suppressed its activation mediated by DFO, underscoring the complexity of this signaling pathway. Our reanalysis of the GSE193817 dataset indicates a significant distinction between quiescent and proliferative smooth muscle cells, suggesting that the dataset primarily captures the transition between these states rather than a straightforward hypoxic response. Although AngII signaling may intersect with the DFO phenotype at the level of specific downstream programs, it does not support a simplistic AngII-rescue model in this context. Overall, this research enhances our understanding of hypoxia and ROS responses in HEsSMCs and emphasizes the potential for personalized medicine strategies informed by sequencing data.

## Data Availability

The datasets generated and/or analyzed during this study are available from the corresponding author upon request.

## Abbreviations

HIF-1α	hypoxia-inducible factor-1 alpha
VHL	von Hippel–Lindau
pVHL	VHL tumor suppressor protein
α-KG	α-ketoglutarate
PHDs	prolyl hydroxylases
NF-κB	nuclear factor-κB
ETC	electron transport chain
ROS	reactive oxygen species
DFO	Desferoxamine
DMOG	Dimethyloxallyl Glycine
AT1R	angiotensin II receptor
ACE	Angiotensin-Converting Enzyme
HEsSMCs	primary human esophageal smooth muscle cells
AngII	Angiotensin II
MTT	thiazolyl blue tetrazolium bromide
PI	propidium iodide
DCFH-DA	2′,7-dichlorofluorescein diacetate
FACS	Fluorescence-Activated Cell Sorting
PE	phycoerythrin
7-AAD	7-Amino-Actinomycin
BrdU	bromodeoxyuridine
AO	acridine orange
MMP	mitochondrial membrane potential
ACTN	α-actinin
GEO	Gene Expression Omnibus
PCA	principal component analysis
GSEA	Gene Set Enrichment Analysis
RAS	renin-angiotensin system
NES	normalized enrichment scores
RAAS	Renin–Angiotensin–Aldosterone System
FIH	factor inhibiting HIF
CDKs	cyclin-dependent kinases
